# Bis(4-nitro­phen­yl) 1,3-phenyl­ene­dimethyl­ene dicarbonate

**DOI:** 10.1107/S1600536808006132

**Published:** 2008-03-12

**Authors:** Syed Nawazish Ali, Sabira Begum, Mitchell A. Winnik, Alan J. Lough

**Affiliations:** a HEJ Research Institute of Chemistry, International Center for Chemical Sciences, University of Karachi, Karachi 75270, Pakistan; b Department of Chemistry, University of Toronto, Toronto, Ontario, Canada M5S 3H6

## Abstract

In the title mol­ecule, C_22_H_16_N_2_O_10_, the dihedral angles between the benzene rings of the 4-nitro­phenyl groups and the central benzene ring are 32.7 (1) and 34.7 (1)°, while the dihedral angle between the two benzene rings of the 4-nitro­phenyl groups is 3.6 (2)°. In the crystal structure, weak inter­molecular C—H⋯O hydrogen bonds link mol­ecules into centrosymmetric dimers.

## Related literature

For related literature, see: Nawazish Ali *et al.* (2008 ▶).
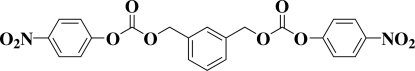


## Experimental

### 

#### Crystal data

C_22_H_16_N_2_O_10_
*M* *_r_* = 468.37
Triclinic, 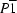

*a* = 8.5956 (4) Å
*b* = 9.2367 (5) Å
*c* = 14.1550 (8) Å
α = 94.094 (2)°
β = 107.134 (3)°
γ = 105.674 (3)°
*V* = 1020.00 (10) Å^3^
*Z* = 2
Mo *K*α radiation
μ = 0.12 mm^−1^
*T* = 150 (1) K
0.34 × 0.20 × 0.20 mm

#### Data collection

Bruker–Nonius KappaCCD diffractometer
Absorption correction: multi-scan (*SORTAV*; Blessing, 1995 ▶) *T*_min_ = 0.808, *T*_max_ = 0.980
10107 measured reflections
4580 independent reflections
3182 reflections with *I* > 2σ(*I*)
*R*_int_ = 0.054

#### Refinement

*R*[*F* ^2^ > 2σ(*F* ^2^)] = 0.064
*wR*(*F* ^2^) = 0.166
*S* = 1.15
4580 reflections
308 parameters
H-atom parameters constrained
Δρ_max_ = 0.30 e Å^−3^
Δρ_min_ = −0.26 e Å^−3^

### 

Data collection: *COLLECT* (Nonius, 2002 ▶); cell refinement: *DENZO–SMN* (Otwinowski & Minor, 1997 ▶); data reduction: *DENZO–SMN*; program(s) used to solve structure: *SIR92* (Altomare *et al.*, 1994 ▶); program(s) used to refine structure: *SHELXTL* (Sheldrick, 2008 ▶); molecular graphics: *SHELXTL*; software used to prepare material for publication: *SHELXTL*.

## Supplementary Material

Crystal structure: contains datablocks global, I. DOI: 10.1107/S1600536808006132/cf2186sup1.cif

Structure factors: contains datablocks I. DOI: 10.1107/S1600536808006132/cf2186Isup2.hkl

Additional supplementary materials:  crystallographic information; 3D view; checkCIF report

## Figures and Tables

**Table 1:** Hydrogen-bond geometry (Å, °)

*D*—H⋯*A*	*D*—H	H⋯*A*	*D*⋯*A*	*D*—H⋯*A*
C10—H10*A*⋯O10^i^	0.95	2.55	3.134 (4)	120
C15—H15*B*⋯O9^i^	0.99	2.58	3.504 (4)	155
C21—H21*A*⋯O7^i^	0.95	2.50	3.264 (3)	138

Symmetry code: (i) 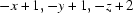.

**Fig. 1.**
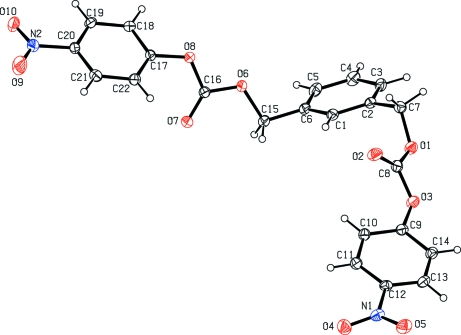
The molecular structure of the title compound showing displacement ellipsoids drawn at the 30% probability level.

**Fig. 2.**
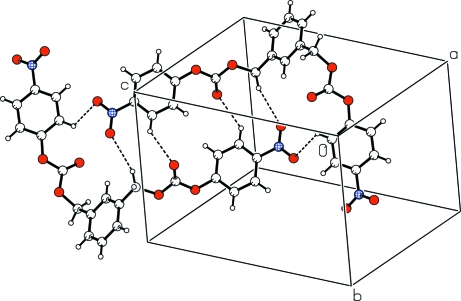
Part of the crystal structure with weak intermolecular C—H···O hydrogen bonds shown as dashed lines.

## supplementary crystallographic information

### Comment

For background information and relevant references see Ali *et al.* (2008). In the title molecule (Fig. 1) the dihedral angles between the benzene rings of the *p*-nitrophenyl groups and the central benzene ring are 32.7 (1) (for C9—C14) and 34.7 (1)° (for C17—C22), while the dihedral angle between the two benzene rings of the *p*-nitrophenyl groups is 3.6 (2)°. In the crystal structure, weak intermolecluar C—H···O hydrogen bonds link molecules into centrosymmetric dimers (Fig. 2).

### Experimental

A solution of 4-nitrophenylchloroformate (14.1 g, 70 mmol) in dry dichloromethane (70 ml) was added dropwise *via* a 250 ml separatory funnel to a solution of 1,3-phenylenedimethanol (4.82 g, 35 mmol) in anhydrous pyridine (5.38 g, 5.5 ml, 68.0 mmol) and dry dichloromethane (20 ml) in a 250 ml round-bottom flask. A white suspension appeared which was stirred gently at room temperature for 10 h. After this time more dry dichloromethane (50 ml) was added, which dissolved the suspension and then the reaction mixture was stirred for another 6 h. It was then quenched by adding deionized water (50 ml). The reaction mixture was transferred to a separatory funnel (500 ml), and the lower organic phase was removed. The aqueous phase was washed with dichloromethane (30 ml *x* 3), and all the dichloromethane solutions were combined. These were then washed with deionized water (40 ml *x* 3), a 1.0% solution of acetic acid (50 ml *x* 4) and once more with deionized water (40 ml *x* 3), and then dried over anhydrous magnesium sulfate and filtered. After filtration, the solvent was removed by rotary evaporator. The product was dried in air overnight in a fume hood and then in a vacuum oven for 24 h at room temperature (< 1 Torr). The desired product was obtained in a moderate yield (11.4 g, 70.0%) as a white solid; the product was recrystallized by dissolving in a mixture of dichloromethane and ethanol (95%) (1:1). The reaction mixture was heated at 358 K, and filtered after 40 minutes. X-ray quality crystals were obtained after slow evaporation of the solvent at room temperature.

### Refinement

Hydrogen atoms were placed in calculated positions with C—H = 0.95–1.00 Å and they were included in the refinement in the riding-model approximation with *U*_iso_(H) = 1.2*U*_eq_(C).

### Crystal data

**Table:**

C_22_H_16_N_2_O_10_	*Z* = 2
*M**_r_* = 468.37	*F*_000_ = 484
Triclinic, *P*1	*D*_x_ = 1.525 Mg m^−^^3^
Hall symbol: -P 1	Mo *K*α radiation λ = 0.71073 Å
*a* = 8.5956 (4) Å	Cell parameters from 10107 reflections
*b* = 9.2367 (5) Å	θ = 2.6–27.5º
*c* = 14.1550 (8) Å	µ = 0.12 mm^−^^1^
α = 94.094 (2)º	*T* = 150 (1) K
β = 107.134 (3)º	Block, colourless
γ = 105.674 (3)º	0.34 × 0.20 × 0.20 mm
*V* = 1020.00 (10) Å^3^	

### Data collection

**Table:**

Bruker–Nonius KappaCCD diffractometer	4580 independent reflections
Radiation source: fine-focus sealed tube	3182 reflections with *I* > 2σ(*I*)
Monochromator: graphite	*R*_int_ = 0.054
Detector resolution: 9 pixels mm^-1^	θ_max_ = 27.5º
*T* = 150(2) K	θ_min_ = 2.6º
φ scans and ω scans with κ offsets	*h* = −11→10
Absorption correction: multi-scan(SORTAV; Blessing, 1995)	*k* = −11→11
*T*_min_ = 0.808, *T*_max_ = 0.980	*l* = −18→18
10107 measured reflections	

### Refinement

**Table:**

Refinement on *F*^2^	Hydrogen site location: inferred from neighbouring sites
Least-squares matrix: full	H-atom parameters constrained
*R*[*F*^2^ > 2σ(*F*^2^)] = 0.064	*w* = 1/[σ^2^(*F*_o_^2^) + (0.035*P*)^2^ + 1.3721*P*] where *P* = (*F*_o_^2^ + 2*F*_c_^2^)/3
*wR*(*F*^2^) = 0.166	(Δ/σ)_max_ < 0.001
*S* = 1.15	Δρ_max_ = 0.30 e Å^−^^3^
4580 reflections	Δρ_min_ = −0.26 e Å^−^^3^
308 parameters	Extinction correction: SHELXTL (Sheldrick, 2008), Fc^*^=kFc[1+0.001xFc^2^λ^3^/sin(2θ)]^-1/4^
Primary atom site location: structure-invariant direct methods	Extinction coefficient: 0.021 (4)
Secondary atom site location: difference Fourier map	

### Special details

**Table:**

Geometry. All e.s.d.'s (except the e.s.d. in the dihedral angle between two l.s. planes) are estimated using the full covariance matrix. The cell e.s.d.'s are taken into account individually in the estimation of e.s.d.'s in distances, angles and torsion angles; correlations between e.s.d.'s in cell parameters are only used when they are defined by crystal symmetry. An approximate (isotropic) treatment of cell e.s.d.'s is used for estimating e.s.d.'s involving l.s. planes.
Refinement. Refinement of *F*^2^ against ALL reflections. The weighted *R*-factor *wR* and goodness of fit *S* are based on *F*^2^, conventional *R*-factors *R* are based on *F*, with *F* set to zero for negative *F*^2^. The threshold expression of *F*^2^ > σ(*F*^2^) is used only for calculating *R*-factors(gt) *etc*. and is not relevant to the choice of reflections for refinement. *R*-factors based on *F*^2^ are statistically about twice as large as those based on *F*, and *R*- factors based on ALL data will be even larger.

### Fractional atomic coordinates and isotropic or equivalent isotropic displacement parameters (Å^2^)

**Table:**

	*x*	*y*	*z*	*U*_iso_*/*U*_eq_	
O1	0.8872 (3)	0.1353 (2)	0.51451 (16)	0.0340 (5)	
O2	0.6463 (3)	0.2045 (3)	0.48906 (18)	0.0406 (6)	
O3	0.8682 (3)	0.3232 (2)	0.43660 (16)	0.0339 (5)	
O4	0.5734 (3)	0.8621 (3)	0.32218 (18)	0.0493 (7)	
O5	0.5700 (3)	0.7631 (3)	0.17823 (17)	0.0453 (6)	
O6	0.7702 (3)	0.1855 (2)	0.94414 (15)	0.0321 (5)	
O7	0.7572 (3)	0.4000 (2)	1.02591 (16)	0.0346 (5)	
O8	0.6017 (3)	0.1596 (2)	1.03074 (15)	0.0304 (5)	
O9	0.1915 (5)	0.4431 (4)	1.2504 (3)	0.0781 (10)	
O10	0.2930 (3)	0.3121 (3)	1.35819 (18)	0.0490 (7)	
N1	0.6000 (3)	0.7673 (3)	0.26870 (19)	0.0343 (6)	
N2	0.2722 (4)	0.3547 (3)	1.2770 (2)	0.0413 (7)	
C1	0.8609 (4)	0.1437 (3)	0.7314 (2)	0.0289 (6)	
H1A	0.7826	0.1949	0.6996	0.035*	
C2	0.9034 (4)	0.0426 (3)	0.6733 (2)	0.0269 (6)	
C3	1.0188 (4)	−0.0314 (4)	0.7203 (2)	0.0326 (7)	
H3A	1.0505	−0.0993	0.6814	0.039*	
C4	1.0875 (4)	−0.0059 (4)	0.8237 (2)	0.0388 (8)	
H4A	1.1658	−0.0571	0.8556	0.047*	
C5	1.0431 (4)	0.0932 (4)	0.8811 (2)	0.0363 (7)	
H5A	1.0893	0.1082	0.9521	0.044*	
C6	0.9314 (4)	0.1712 (3)	0.8356 (2)	0.0313 (7)	
C7	0.8208 (4)	0.0058 (3)	0.5615 (2)	0.0333 (7)	
H7A	0.8443	−0.0854	0.5354	0.040*	
H7B	0.6954	−0.0173	0.5444	0.040*	
C8	0.7845 (4)	0.2182 (3)	0.4820 (2)	0.0300 (6)	
C9	0.7965 (4)	0.4351 (3)	0.3983 (2)	0.0288 (6)	
C10	0.7170 (4)	0.5092 (3)	0.4481 (2)	0.0311 (7)	
H10A	0.7052	0.4839	0.5100	0.037*	
C11	0.6548 (4)	0.6215 (3)	0.4058 (2)	0.0314 (7)	
H11A	0.6008	0.6758	0.4387	0.038*	
C12	0.6728 (4)	0.6530 (3)	0.3153 (2)	0.0287 (6)	
C13	0.7562 (4)	0.5817 (4)	0.2664 (2)	0.0320 (7)	
H13A	0.7691	0.6080	0.2049	0.038*	
C14	0.8204 (4)	0.4712 (3)	0.3092 (2)	0.0310 (7)	
H14A	0.8799	0.4208	0.2781	0.037*	
C15	0.8850 (4)	0.2793 (4)	0.8979 (3)	0.0382 (8)	
H15A	0.9887	0.3477	0.9501	0.046*	
H15B	0.8267	0.3424	0.8556	0.046*	
C16	0.7147 (4)	0.2644 (3)	1.0016 (2)	0.0269 (6)	
C17	0.5156 (4)	0.2113 (3)	1.0894 (2)	0.0261 (6)	
C18	0.5105 (4)	0.1426 (3)	1.1723 (2)	0.0284 (6)	
H18A	0.5621	0.0642	1.1865	0.034*	
C19	0.4293 (4)	0.1892 (3)	1.2346 (2)	0.0295 (6)	
H19A	0.4271	0.1461	1.2933	0.035*	
C20	0.3518 (4)	0.2999 (3)	1.2088 (2)	0.0294 (6)	
C21	0.3483 (4)	0.3636 (3)	1.1233 (2)	0.0312 (7)	
H21A	0.2898	0.4368	1.1067	0.037*	
C22	0.4322 (4)	0.3182 (3)	1.0620 (2)	0.0279 (6)	
H22A	0.4323	0.3597	1.0025	0.033*	

### Atomic displacement parameters (Å^2^)

**Table:**

	*U*^11^	*U*^22^	*U*^33^	*U*^12^	*U*^13^	*U*^23^
O1	0.0368 (12)	0.0373 (12)	0.0328 (11)	0.0141 (10)	0.0145 (10)	0.0116 (9)
O2	0.0349 (13)	0.0401 (13)	0.0535 (15)	0.0122 (10)	0.0215 (11)	0.0171 (11)
O3	0.0352 (12)	0.0365 (12)	0.0347 (12)	0.0114 (10)	0.0163 (10)	0.0116 (10)
O4	0.0688 (18)	0.0461 (15)	0.0451 (14)	0.0304 (14)	0.0230 (13)	0.0126 (12)
O5	0.0525 (15)	0.0504 (15)	0.0315 (12)	0.0159 (12)	0.0094 (11)	0.0155 (11)
O6	0.0388 (12)	0.0276 (11)	0.0331 (11)	0.0059 (9)	0.0210 (10)	0.0018 (9)
O7	0.0409 (12)	0.0249 (12)	0.0371 (12)	0.0039 (9)	0.0185 (10)	0.0001 (9)
O8	0.0381 (12)	0.0240 (11)	0.0351 (11)	0.0082 (9)	0.0217 (10)	0.0060 (9)
O9	0.110 (3)	0.081 (2)	0.102 (3)	0.070 (2)	0.078 (2)	0.0417 (19)
O10	0.0583 (16)	0.0476 (15)	0.0417 (14)	0.0040 (12)	0.0308 (13)	−0.0023 (11)
N1	0.0335 (14)	0.0357 (15)	0.0321 (14)	0.0068 (12)	0.0105 (12)	0.0111 (12)
N2	0.0466 (17)	0.0318 (15)	0.0512 (18)	0.0068 (13)	0.0302 (15)	0.0010 (13)
C1	0.0275 (15)	0.0283 (15)	0.0332 (16)	0.0083 (12)	0.0135 (13)	0.0064 (13)
C2	0.0259 (15)	0.0261 (15)	0.0294 (15)	0.0060 (12)	0.0114 (12)	0.0056 (12)
C3	0.0328 (16)	0.0361 (17)	0.0366 (17)	0.0152 (14)	0.0180 (14)	0.0077 (14)
C4	0.0333 (17)	0.050 (2)	0.0390 (18)	0.0189 (16)	0.0137 (15)	0.0152 (16)
C5	0.0319 (16)	0.047 (2)	0.0261 (15)	0.0045 (15)	0.0105 (13)	0.0049 (14)
C6	0.0306 (16)	0.0287 (16)	0.0349 (16)	0.0022 (13)	0.0182 (14)	0.0026 (13)
C7	0.0425 (18)	0.0285 (16)	0.0299 (16)	0.0112 (14)	0.0127 (14)	0.0058 (13)
C8	0.0357 (17)	0.0294 (16)	0.0235 (14)	0.0092 (13)	0.0087 (13)	0.0034 (12)
C9	0.0308 (15)	0.0265 (15)	0.0260 (14)	0.0053 (12)	0.0076 (12)	0.0051 (12)
C10	0.0377 (17)	0.0299 (16)	0.0229 (14)	0.0059 (13)	0.0103 (13)	0.0022 (12)
C11	0.0341 (16)	0.0290 (16)	0.0280 (15)	0.0048 (13)	0.0106 (13)	0.0017 (12)
C12	0.0284 (15)	0.0261 (15)	0.0265 (15)	0.0031 (12)	0.0059 (12)	0.0051 (12)
C13	0.0312 (16)	0.0350 (17)	0.0256 (15)	0.0014 (13)	0.0108 (13)	0.0068 (13)
C14	0.0279 (15)	0.0330 (17)	0.0302 (15)	0.0047 (13)	0.0113 (13)	0.0041 (13)
C15	0.0449 (19)	0.0300 (17)	0.0402 (18)	0.0003 (14)	0.0262 (16)	0.0001 (14)
C16	0.0280 (15)	0.0261 (16)	0.0242 (14)	0.0051 (12)	0.0085 (12)	0.0025 (12)
C17	0.0284 (15)	0.0236 (14)	0.0251 (14)	0.0046 (12)	0.0112 (12)	−0.0005 (11)
C18	0.0316 (16)	0.0245 (15)	0.0301 (15)	0.0077 (12)	0.0118 (13)	0.0067 (12)
C19	0.0314 (16)	0.0290 (16)	0.0276 (15)	0.0038 (13)	0.0132 (13)	0.0067 (12)
C20	0.0299 (15)	0.0250 (15)	0.0330 (16)	0.0028 (12)	0.0164 (13)	−0.0021 (12)
C21	0.0288 (15)	0.0277 (16)	0.0362 (17)	0.0088 (13)	0.0097 (13)	0.0032 (13)
C22	0.0300 (15)	0.0277 (15)	0.0229 (14)	0.0067 (12)	0.0063 (12)	0.0037 (12)

### Geometric parameters (Å, °)

**Table:**

O1—C8	1.324 (4)	C5—H5A	0.950
O1—C7	1.472 (4)	C6—C15	1.494 (4)
O2—C8	1.195 (4)	C7—H7A	0.990
O3—C8	1.362 (3)	C7—H7B	0.990
O3—C9	1.404 (3)	C9—C10	1.379 (4)
O4—N1	1.227 (3)	C9—C14	1.385 (4)
O5—N1	1.225 (3)	C10—C11	1.385 (4)
O6—C16	1.323 (3)	C10—H10A	0.950
O6—C15	1.467 (3)	C11—C12	1.377 (4)
O7—C16	1.199 (3)	C11—H11A	0.950
O8—C16	1.355 (3)	C12—C13	1.382 (4)
O8—C17	1.400 (3)	C13—C14	1.382 (4)
O9—N2	1.219 (4)	C13—H13A	0.950
O10—N2	1.220 (4)	C14—H14A	0.950
N1—C12	1.468 (4)	C15—H15A	0.990
N2—C20	1.471 (4)	C15—H15B	0.990
C1—C2	1.391 (4)	C17—C18	1.380 (4)
C1—C6	1.394 (4)	C17—C22	1.383 (4)
C1—H1A	0.950	C18—C19	1.386 (4)
C2—C3	1.392 (4)	C18—H18A	0.950
C2—C7	1.501 (4)	C19—C20	1.379 (4)
C3—C4	1.384 (4)	C19—H19A	0.950
C3—H3A	0.950	C20—C21	1.378 (4)
C4—C5	1.381 (4)	C21—C22	1.389 (4)
C4—H4A	0.950	C21—H21A	0.950
C5—C6	1.390 (4)	C22—H22A	0.950
			
C8—O1—C7	116.3 (2)	C9—C10—H10A	120.7
C8—O3—C9	119.8 (2)	C11—C10—H10A	120.7
C16—O6—C15	114.3 (2)	C12—C11—C10	118.7 (3)
C16—O8—C17	118.4 (2)	C12—C11—H11A	120.7
O5—N1—O4	123.6 (3)	C10—C11—H11A	120.7
O5—N1—C12	117.9 (3)	C11—C12—C13	122.9 (3)
O4—N1—C12	118.5 (2)	C11—C12—N1	118.5 (3)
O9—N2—O10	123.2 (3)	C13—C12—N1	118.6 (3)
O9—N2—C20	118.2 (3)	C12—C13—C14	118.4 (3)
O10—N2—C20	118.6 (3)	C12—C13—H13A	120.8
C2—C1—C6	121.0 (3)	C14—C13—H13A	120.8
C2—C1—H1A	119.5	C13—C14—C9	118.8 (3)
C6—C1—H1A	119.5	C13—C14—H14A	120.6
C1—C2—C3	119.2 (3)	C9—C14—H14A	120.6
C1—C2—C7	121.4 (3)	O6—C15—C6	106.4 (2)
C3—C2—C7	119.3 (3)	O6—C15—H15A	110.4
C4—C3—C2	119.9 (3)	C6—C15—H15A	110.4
C4—C3—H3A	120.0	O6—C15—H15B	110.4
C2—C3—H3A	120.0	C6—C15—H15B	110.4
C5—C4—C3	120.6 (3)	H15A—C15—H15B	108.6
C5—C4—H4A	119.7	O7—C16—O6	128.0 (3)
C3—C4—H4A	119.7	O7—C16—O8	126.2 (3)
C4—C5—C6	120.4 (3)	O6—C16—O8	105.7 (2)
C4—C5—H5A	119.8	C18—C17—C22	122.3 (3)
C6—C5—H5A	119.8	C18—C17—O8	116.0 (2)
C5—C6—C1	118.8 (3)	C22—C17—O8	121.6 (2)
C5—C6—C15	120.2 (3)	C17—C18—C19	119.2 (3)
C1—C6—C15	120.9 (3)	C17—C18—H18A	120.4
O1—C7—C2	110.4 (2)	C19—C18—H18A	120.4
O1—C7—H7A	109.6	C20—C19—C18	118.0 (3)
C2—C7—H7A	109.6	C20—C19—H19A	121.0
O1—C7—H7B	109.6	C18—C19—H19A	121.0
C2—C7—H7B	109.6	C21—C20—C19	123.2 (3)
H7A—C7—H7B	108.1	C21—C20—N2	118.6 (3)
O2—C8—O1	128.1 (3)	C19—C20—N2	118.2 (3)
O2—C8—O3	126.4 (3)	C20—C21—C22	118.5 (3)
O1—C8—O3	105.5 (2)	C20—C21—H21A	120.8
C10—C9—C14	122.6 (3)	C22—C21—H21A	120.8
C10—C9—O3	122.7 (3)	C17—C22—C21	118.6 (3)
C14—C9—O3	114.6 (3)	C17—C22—H22A	120.7
C9—C10—C11	118.5 (3)	C21—C22—H22A	120.7

### Hydrogen-bond geometry (Å, °)

**Table:**

*D*—H···*A*	*D*—H	H···*A*	*D*···*A*	*D*—H···*A*
C10—H10A···O10^i^	0.95	2.55	3.134 (4)	120
C15—H15B···O9^i^	0.99	2.58	3.504 (4)	155
C21—H21A···O7^i^	0.95	2.50	3.264 (3)	138

Symmetry codes: (i) −*x*+1, −*y*+1, −*z*+2.
